# Reasons for the Place of Care of the Elders: A Systematic Review

**DOI:** 10.3390/healthcare8040436

**Published:** 2020-10-27

**Authors:** Gema Serrano-Gemes, Manuel Rich-Ruiz, Rafael Serrano-del-Rosal

**Affiliations:** 1 Institute for Advanced Social Studies-Spanish National Research Council (IESA-CSIC), 14004 Córdoba, Spain; rserrano@iesa.csic.es; 2 Maimonides Biomedical Research Institute of Cordoba (IMIBIC), University of Cordoba (UCO), Reina Sofia University Hospital (HURS), 14004 Córdoba, Spain; en1rirum@uco.es; 3 CIBER on Frailty and Healthy Ageing (CIBERFES), 28029 Madrid, Spain

**Keywords:** decision-making, push-pull factors, location of care, older people, reasons

## Abstract

Background: Relocation is a very important event in people’s lives in general, but really significant in old age. However, some predictors of relocation still need to be improved. The objective of this review was to synthesize qualitative evidence to understand the reasons of the participants to decide on the place of care of the older people. Methods: Systematic review of qualitative studies was conducted in six databases: Scopus, SciELO, PubMed, PsycINFO, Web of Science and CINAHL, from its beginning until 29 November 2017. Qualitative or mixed studies, written in English or Spanish and addressing the decision-making process (already experienced by participants) on the place of care of older persons (65 years or older), were included in the review. PROSPERO (registration number: CRD42018084826). Results: A total of 46 articles were finally included in the analysis. Our main result is the distinction of multiple reasons for each population group involved in the decision-making process, ranking these reasons into three factors: Retention, pull and push. Conclusion: This differentiation allows for a more detailed and in-depth analysis of the motivations of the different groups involved in this process.

## 1. Introduction

Many studies and theories focusing on the study of migrations over the years are available [1]. Migrations can be defined as a displacement or change of housing, which must be of a certain permanent nature, or at least, it should possess intention of permanence, and this change is performed at a significant distance [2]. The model derived from the well-known theory of push-pull is the migratory explanatory model that has had a greater impact in the scientific field [1]. 

The analytical framework of “push” and “pull” factors is widely known, being used for the first time, implicitly, by Ravenstein in 1885, and it was later analyzed by Lee in 1966 [2]. Lee pointed to different factors involved in the migration decision: factors related to the place of origin, factors related to the place of destination, problems, and factors of a personal nature [3]. This author highlighted that both the place of origin and the place of destination have factors maintaining people in the place or attracting them, and factors that push them out [3].

From a motivational point of view, empirical evidence often distinguishes between push and pull factors in the relocation process [4]. These authors stated that despite the fact these dichotomous factors are useful, they can be enriched by taking into account the motivations leading to relocation [4]. There is also the need to refine certain predictors of relocation [4]. 

In addition, it is important to note that relocation is understood as a significant event at the emotional level at any time in a person’s life, but especially in old age [4].

This fact is even more relevant if we take into account the projections of an increase in the number of older people, thus leading to an aging of the global population [5]. In this regard, the United States Department of Health and Human Services, in a report related to long-term care, already pointed out a few years ago that the increase in demand for long-term care services in the coming years would be mainly related to the aging of the baby boom generation [6]. Additionally anticipating in this same report that an intensification of long-term service requests in both community and institutional care settings for the older people would occur [6].

However, as shown in the World Report on Aging and Health, although evidence suggests that placing older people at the center of healthcare is the best way to respond to their needs, health services at the global level are still not in line with the health problems of this group [7]. This deficient attention is hampered by both the lack of knowledge of the needs and priorities of the older people, and the discrimination suffered by the elders in the different health services, for example, not being asked about their preferences regarding their care [7].

In fact, one of the three basic principles of evidence-based medicine is that evidence is not the only element necessary to make a clinical decision [8]. In addition to the skills of professionals in interpreting the patient’s situation and the available evidence, clinical decisions must also be consistent with the preferences and values reported by the patient [8]. 

However, shared decision-making is not commonly used despite all its benefits [9]. Today, little consensus and research on decision-making preferences of elders regarding their health is found [10].

Due to all of the above, the research question in this article was: What are the participants’ reasons to decide on the place of care of the older people? Our objective being to synthesize the evidence with qualitative methodology to understand the reasons for participants being involved in the decision-making process on the place of care of the older people.

This review arises from a wider systematic review dealing with the decision-making process on the place of care for the older people by taking into account different aspects of interest: the participants in the process, their experiences and their reasons. The protocol for this wider systematic review was registered in PROSPERO (registration number: CRD42018084826), and it is already published [11]. Our group has recently published a study addressing the first of these aspects, that is, the participants [12]. The present review focuses on the third of these aspects, that is, the reasons of the participants.

## 2. Methods

### 2.1. Design

We conduct a systematic review of qualitative studies. This review was informed by Enhancing transparency in reporting the synthesis of qualitative research (ENTREQ) statement [13] and Preferred Reporting Items for Systematic Reviews and Meta-Analyses (PRISMA) statement [14] (Supplementary Materials: Table S1: PRISMA Checklist).

In this study, the following inclusion criteria were used: (a) original studies published in English or Spanish with qualitative or mixed-method; (b) studies dealt with the decision-making process on the place of care (already experienced by the participants) of the older people, adults who are 65 years or older.

The following studies were excluded from the present review: (a) studies about decisions on the place to die, palliative care, terminal patient care, advance directives, advance care planning, and/or the end of life; (b) studies about decisions on acute care, specific health problems (such as psychiatric inpatient care) and/or temporary places of care; (c) studies where relocation has started in an institutional environment; (d) studies about decisions on the place of care related to intellectual disabilities or substance abuse; (e) conference proceedings (conference abstracts) or doctoral theses; (f) studies whose complete text was not accessible.

More information about the inclusion and exclusion criteria of studies can be checked in the review protocol [11].

### 2.2. Search Strategies 

To ensure an exhaustive and specific search, we developed, refined and conducted the strategy in different databases: Scopus, core collection of Web of Science, SciELO Citation Index (through Web of Science), MEDLINE (through PubMed), PsycINFO (through ProQUEST), and CINAHL Complete (through EBSCOhost). 

These searches in the databases were performed from the beginning of the databases until 29 November 2017.

These searches focused on five key concepts: (1) elders, (2) relocation/location of care, (3) different options of places of care, (4) decision making, and (5) issues not related to the object of the study but which could confuse the search strategy: intellectual disability, substance abuse, place to die, palliative care, terminal patient care, advance directives, advance care planning, and the end of life (which are composed of different terms). The terms that composed each concept were linked using the OR connector. Later, the first four concepts were joined using the AND connector, while the fifth was joined to the others using the NOT connector. Finally, this strategy was adapted to each database by using its specific terminology to adjust to the requirements of each one of them. The different search strategies can be found in a previously published review protocol [11].

### 2.3. Article Selection 

To determine inclusion and exclusion criteria of studies obtained in the search strategy, two reviewers independently examined the title and abstract of each article. In cases of disagreement, the third reviewer intermediated. Then, the same process was performed with the full-text articles.

**Figure 1:**
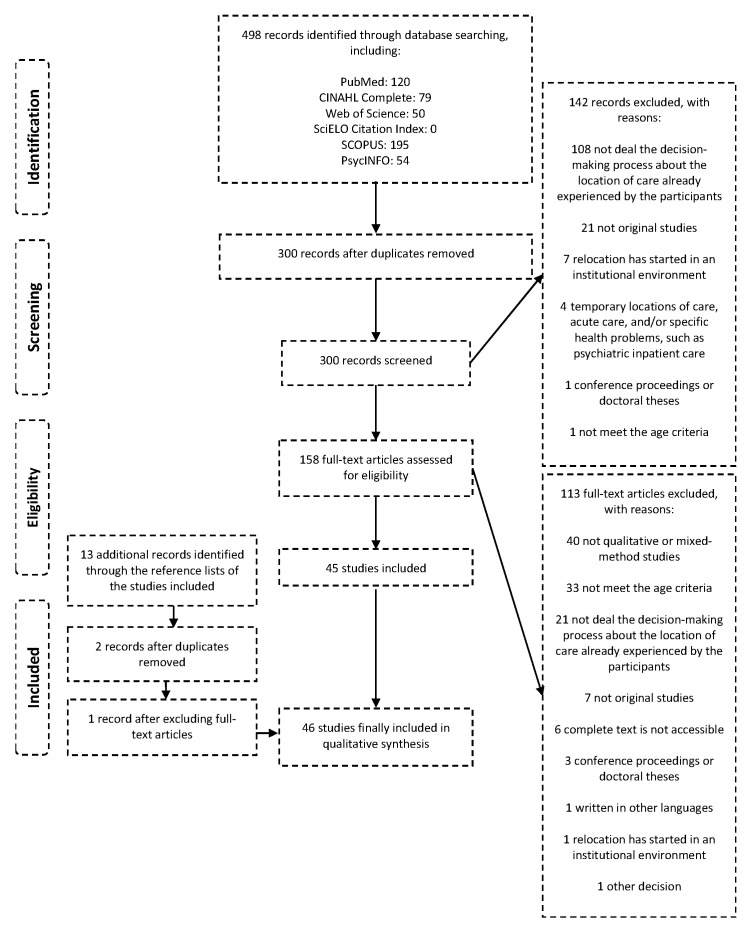
Flow chart (Modified from the Preferred Reporting Items for Systematic Reviews and Meta-Analyses (PRISMA) Flow Diagram [14]). Source: Own elaboration based on the information obtained. More information about the identification in the review protocol [11].

In order to include in the review all articles of interest related to the topic, the list of references in the papers finally included were also reviewed. Figure 1 shows the complete process of selection of articles.

### 2.4. Data Extraction

A tool specifically developed for the purpose of this study was used for the extraction of data from the finally included articles. This tool had two parts: one descriptive, while the other one is directly related to the objective of the review.

Information was collected on the first part of the tool: title, year of publication, country, authors, objective of the study, design/methodological basis, sample, techniques/methods, and data analysis methods/techniques. This information is summarized in Table 1.

The second part of the tool focused on: results and the final conclusion. This information obtained in this second part of the tool was subsequently analyzed in depth in the results section.

### 2.5. Quality Appraisal

In this review, a template to help understand a qualitative study designed by the Critical Appraisal Skills Programme Español (CASPe) [15], in particular, the Spanish version, was used.

The rating and scoring system suggested by Butler et al. [16] has been used to score the quality level of the various studies included: YES = 1, NOT SURE = 0.5, and NO = 0. These authors established three categories based on the score obtained: high-quality (Scores 9–10), moderate-quality (Scores 7.5–9), low-quality (less than 7.5), and excluded those with less than 6 points. However, in the present review the quality of the studies was not considered an inclusion/exclusion criterion; therefore, no studies have been excluded based on their quality.

Two reviewers independently assessed the quality of the articles finally included in the review, discussing with a third reviewer the cases in which discrepancies were found to reach an agreement.

The relative contribution of the articles to our results, according to their quality, has also been analyzed considering a process previously proposed by other authors [17]. In this review, each time a study provided information to the results was considered a relative contribution. Thus, a score of 7 means that the study has contributed seven times to the results of the present review. 

Table 1 shows information on the quality assessment, the score and the relative contribution of the articles. 

### 2.6. Data Analysis

We synthesized the data of included studies using the Constant Comparative Method [18] from Glaser and Strauss’ Grounded Theory [19]. 

We conducted the following steps to synthesize the findings of the included studies. First, we read and reread the results and the final conclusions of the different studies to understand in depth their meanings. Second, we classified the information using a widely used and known classification of reasons: pull and push factors (taking into account the definitions suggested by Lee [3]). At all times, the possibility of emerging categories was considered; in addition, the constant comparison of the created categories and subcategories was performed. 

Two authors performed the analysis of data independently, and the third author was asked when needed. Finally, all authors reviewed and discussed the results ensuring that they fitted the original information. 

## 3. Results

### 3.1. Study Characteristics, Quality and Contribution

A total of 498 articles were identified through our search strategy in the different databases [11]. Additionally, 13 articles were identified after reviewing the reference list of the studies finally included. Finally, 46 articles were included in this systematic review (Figure 1). 

Of the 300 articles reviewed, there were only 15% discrepancies between the two reviewers, in which the third author was consulted in order to reach an agreement. Therefore, the percentage of agreement was higher than 80%.

The 46 studies included were conducted in different countries, although the vast majority were conducted in the United States. The rest of the countries, from largest to smallest number of studies contributed, were: Canada, Sweden, New Zealand, Australia, Finland, Taiwan, United Kingdom, China, Germany, Israel, Japan, Norway, and South Korea. 

Regarding quality, most of the articles included have obtained a moderate quality (22 articles), 16 articles obtained a high quality, and 8 articles obtained a low quality.

Regarding the relative contribution of the studies, no relationship was found between the relative contribution of the studies to the results of the review and the quality of the articles. This lack of correlation occurs because some articles have obtained a great contribution and they have shown a low quality, such as the study of Gottlieb et al. [20]; in contrast, low contribution and high quality was found, for example, in the study conducted by Wilson et al. [21]. 

The study with the highest relative contribution was conducted by Ducharme et al. [22] (with a high quality); whereas the study conducted by Johnson et al. [23] (with moderate quality) obtained the lowest contribution. 

Table 1 shows more details.

### 3.2. Key Findings

Some studies included in our literature review [35,40,46,50] classify the reasons causing older people to move into two categories: push and pull factors. 

However, in this review, many reasons have been found that did not fit this well-known dual classification, responding much better to a three-factor classification, which simply arises from subdividing the “pull” factor into two (based on the definition suggested by Lee [3]), understanding that there may be “retention/maintenance” factors at the place of origin, “push” from the place of origin, and “pull” to the new place. 

Thus, in this review, the reasons mentioned in the different studies included in our results have been classified, first, in factors of: retention, pull or push. Second, within each of these factors, the reasons have been subclassified into thematic categories. Third, each factor and category has been explained by the reasons constituting them (showing the factors mentioned in a larger number of studies included in the revision), and those accompanied by different quotations (Q) on some occasions (Supplementary Materials: Text S1: Original Quotes).

Finally, we performed a further subclassification according to the different participants that have been found in this decision-making process, following the classification presented in another recently published systematic review: older people, family members, professionals and other relevant people, such as friends and neighbors [12]. 

Accordingly, it was possible to analyze the results found in this review in a more thorough and complete manner, and to focus in a much more precise way on the studied object: the decision about the place of care, in any of its options (not only the decision to move, but also, for example, the decision of not relocating). 

**Figure 2:**
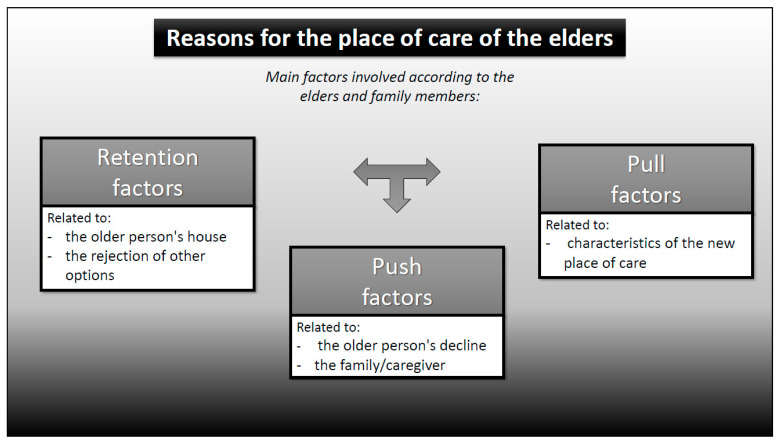
Main factors involved according to the elders and family members. Source: Own elaboration based on the information obtained from the studies included in this systematic review (more detailed information on these factors can be found in the Supplementary Materials: Table S2: Main factors: elders and family members).

A brief summary of our results can be seen in Figure 2.

### 3.3. Retention Factors

Retention factors have been understood in this review as all factors making the older persons not change the place of care in which they are located. 

These factors have been mentioned by all studied population groups: older people, family, professional and other relevant persons. However, there are differences among them.

Thus, although the categories constituting the retention factor in older people and family members coincide (“the house”, “the rejection of other options” and “facilitating elements”), the specific reasons constituting them are, on certain occasions, different/specific and even unique according to the population group.

For example, the “own house” category shows how older people want to be at home [25,27,36,41,42,52], maintain autonomy (freedom and privacy) [51,64], maintain roles and routines [51], or maintain their social networks [51,64], finding that some older people have a strong emotional bond with the home [51,64]. 

Furthermore, the family members mention completely different aspects in the category of “house of the older person”. They emphasize their desire to care at home [32,36,41,42,43,59], while reflecting on the health status of the older person [22,41]. However, they also mention the difficulty in deciding for the older person [22] and even the loneliness they would feel if the older person was not with them [22,34] as reasons.

Another example would be the category of “rejection of other options”. Although this category has been mentioned by both older people and family members, some differences have been found between these two groups. Older people refer to different kinds of reasons making them reluctant to change a place of care, such as reluctance to control [45], to use home help and/or technical help [52,62] or to live in other places [20,45,62]; the relatives, on the other hand, among their reasons of “rejection of other options”, sometimes mention the refusal of the older person [22,32,43,49,64] and the refusal of the professionals [48].

Regarding the facilitators, although the content of these does not differ too much, since both population groups mention the support of other people and the environment is adequate, older people mention specific facilitators related to their own health and capacity (be still too healthy [63]; feeling well [22,51,52,62]; or mobility of the older person [42]). 

More details are found in Supplementary Materials: Table S2: Main factors: elders and family members.

Moreover, the group of professionals and the group of other relevant people differ in greater measure from the other groups. 

For professionals, within the retention factor, the main category to consider is that relocation is not necessary [36,48], so they choose to delay relocation [48], for example, because of the possibility of causing confusion and distress in the patient [48]. 

However, sometimes professionals decide not to change the place of care because the older person has family support [32] (Q13) [32]. Although, on other occasions, they simply make this decision to satisfy the family [32] (Q14) [32] or to preserve the perception of independence of the older person [32], these latter reasons being consistent, in a certain way, with those expressed by the older people and family. 

Finally, for the group of other relevant people, the only category and reason expressed to retain the older person in the current environment has been to consider that older people were too young to move [20,35] (Q15) [35]. This resembles, in some way, some of the older people’s facilitators. 

### 3.4. Pull Factors

In this review, the pull factors have been interpreted as all those reasons that pull towards a new place of care.

This type of factor has only been mentioned by older people and family members. Categories directly mentioning a new place of care, that is, those reasons directly related to the new place of care and the characteristics of this place, were the most often mentioned by these two groups. Most of the reasons constituting these categories are consistent between both groups, only sometimes we found specific and particular aspects for one of the groups. An example of a difference would be the reason “familiarity and reputation” [20,25,35,40,45,47,50,62] reported by older people, a reason not reported by family members, although these mention a related aspect, such as “quality of the place of care” [30]. Another example in which certain differences were found would be the reason of “social environment” [20,35,45,46,47,50,53] reported by older people, in which the importance of having “opportunities for socialization” [20,35,45,46,53] or “living with people of the same age” [46] is highlighted. In contrast, family members have no equivalent reason, although they are somewhat concerned about aspects related in one way or another to the social environment of the older person, for example, mentioning the importance of taking into account the “staff of these places” [36,64] and the “status and behavior of the residents of the places of care” [64]. 

Supplementary Materials: Table S2: Main factors: elders and family members show further details on the main pull factors of elders and family members. 

Finally, both older people and family members mentioned other general pull categories, not directly related to a place of care, but simply reasons that encouraged them to change, and to go to other places of care. For the older people, the following pull factors were found: temporal access to relocation [36,39,62], seek independence [20,37], importance of information [45,55], avoid further movings [20], seek a new lifestyle and activities [46], have a new partner [51], seek security [40], and receive advice from others [53]. In contrast, for family members, these general pull categories are: Information on places of care [26,30,64] (Q26) [26], care needed for older person [29,34], search for a safer environment [29,34], increase the social network of the older person [22], preserve the independence of older people [32], and improve the elder’s quality of life [34].

### 3.5. Push Factors

Push factors have been understood as all those factors leading the elders to change place of care, pushing them out and/or encouraging them to leave the current place. 

Older people, family members and professionals have mentioned, to a greater or lesser extent, reasons for this type of factor.

The categories mentioned by older people and family members are, at first glance, quite similar, although they have certain differences.

Both groups mention the “decline of the older people” category, highlighting the deterioration of health of older people and their need for help. However, family members report about “falls” [22,30,31,32,34,36,38,41,42,48,58] as a relevant reason, while this is not found among the most mentioned category in the group of older people. Older people often mention “isolation/loneliness” [20,24,32,40,46,53,54,61,63] and “insecurity/unsafety” [23,32,36,45,56,60,61,63,64] as relevant reasons, while relatives do not mention them so frequently.

Despite both the elders and their relatives mentioning problems related to the burden of care, older people reported family-related reasons in general, while family members reported more specifically problems directly related to the caregivers. Another factor showing differences is both groups mention the environment, family members reported reasons related to both the formal and informal environment, whereas older people referred only to the formal environment.

Finally, these differences between the two groups are even more obvious if we look at the categories mentioned by the group of older people. The latter group have a unique category, such as: “Anticipate” [20,24,35,46,52,53,60,61,63]: whether acting in advance [24,46,52,53,63], taking into account previous experiences of known people [20,24,35] or being the timely moment [35,53,60]. In contrast, family members do not point this out among their most frequently mentioned reasons.

Supplementary Materials: Table S2: Main factors: elders and family members show further details on main push factors of elders and family members.

Furthermore, the push categories reported by the professionals [22,25,27,30,31,32,38,41,42,43,45,48,54,60,63,65] are mainly two.

The first push category reported by the professionals was their concern about the elders [25,27,30,32,38,41,42,45,48,63,65], which contemplates: the elders’ deterioration [25,27,38,41,42,63], their fragility [32], their loneliness [41], their low quality of life [41], their safety [32,41,65], their well-being [25,30,45], the improvement of the quality of care [48], and because the elder had the necessary level of care [41]. 

Whereas the second push category would be their concern for the caregiver [22,27,41,43,48], a category that focuses on: protecting the physical and psychological health of the caregivers [48], concern about their exhaustion [41,43], and the fact that they have reached the point where the caregiver needs to relocate the older person [22,27,43].

However, professionals also have other categories. Whether they are reasons related to the place where the older person lives [22,48,63]: because, for example, the home does not adapt to the needs of the older person [63], or because the current place of care can no longer provide the required care [22]; or related to the hospital [30,31,54,60]: stopping hospital care and thus discharging the older person [30,31], suggesting from the hospital the relocation [30,54] to avoid new admissions [54], and even, prompting the professionals themselves the relocation during the hospitalization [60]. 

Although professionals reported categories, which a priori may seem different from those reported by older persons and family members, the reasons constituting these categories are quite similar to the reasons mentioned by the other population groups. 

## 4. Discussion

The main outcome of the review has been the existence of a wide range of reasons involved in the relocation decision-making process in the different population groups analyzed. Along with this large variety, another important result has been the yield of a new categorization of reasons in three factors (retention, pull and push), which has allowed to make a more detailed and in-depth analysis of this process. 

More specifically, the retention categories found have been multiple, especially in the group of older people and family members. However, the categories mentioned most often by these two groups were those related to the retention caused by the house, as well as the rejection of other options. In relation to this latter aspect, and more specifically to the reluctance to control expressed by older people in our review, another study of the literature shows similar results [66], with quantitative methodology, which highlights how the perception of loss of independence and lack of privacy were mostly informed by older adults as an aspect that is likely to discourage relocation. 

Among the retention categories of the professionals found in our review, it is noteworthy that they consider that the relocation of the older person is not necessary. However, sometimes, although the professionals did not want the older person to live alone, they allowed it because of the family support available to the elder. This latter aspect is of particular interest because it is consistent directly with the systematic review of Friedman et al. [67]. The mentioned review looked at whether family caregivers offset healthcare costs for older people, showing as part of their results that having family caregivers delays or decreases older people’s entry into a nursing home [67]. Thus, having family support seems to make staying at home easier for the older people, thereby delaying entry into an institutional environment. 

Furthermore, within the group of other relevant people, older people’s friends pointed out that they do not find the location necessary, highlighting that they were still too young to make that kind of decision, which is consistent with the results of the study developed by Crisp et al. [66], in which some older adults pointed out that they perceived the retirement villages as places only suitable for older people (although it was a factor mentioned in less proportion than other factors). 

In relation to the pull factor, it is interesting to point out two aspects of our results. First, only two groups have provided reasons for this type of factor: older people and family members. The second aspect of interest is that, in contrast with the other two factors (retention and push), which were composed of a greater variety of categories with multiple reasons, the pull factor was focused mainly on one single category: characteristics of the new place of care. Thus, this category of our review is consistent with the quantitative study of Crisp et al. [66], who indicate that a large part of the participants, in relation to the characteristics of the retirement village, agreed about having some kind of independence, having space to be able to take a walk, a factor of assisted living and access to medical facilities, as significant elements influencing relocation decisions.

Finally, with regard to push factor, three population groups have provided information: older people, family members and professionals. For all of them, the most supported categories were those with aspects related to the older person and the family/caregiver. With regard to the category related to the older person, the reasons have focused mainly on the deterioration of the health of the older person and the need for assistance of the older person, which is consistent with the review of Henkens et al. [68], who reported that the greater reliance on support services and health deterioration are two essential elements motivating the decisions of housing relocation. 

Moreover, regarding the push categories, we found in our review that the category considering aspects related to the caregivers, highlighting how family members feel unable to continue the care and the deterioration of their own state of health, has been reported quite frequently. This result is consistent with the quantitative study of Buhr et al. [69], who reported that the most frequent reasons given by caregivers for institutionalization were that their loved ones needed a more complex care than that they could provide to the elder and that the caregivers’ own health would make it impossible to continue performing the care tasks.

### Strengths and Limitations

The strengths of the present study should be highlighted. The results of this review represent a significant contribution to the international literature on the reasons arising throughout the decision-making process on the place of care of elders. 

Previous studies, such as the systematic review of Roy et al. [70], have addressed the factors involved in housing decisions among frail older adults. However, the significance of the present review is because it complements and helps to develop this field of research by taking into account all the people involved in the process. Additionally, we have performed an in-depth qualitative analysis, which has allowed us to analyze the studied process more closely. 

Therefore, we believe that all this information will make it possible to make improvements not only at the organizational level or for the improvement of the work and approach of the professionals facing every day this decision-making process, but also that the older people themselves will benefit, as well as their nearest environment, because their needs and preferences are recognized in this process.

In addition, we would also like to point out what this article brings in relation to our previous publications on this research topic. As noted in the introduction section above, this review addresses the reasons given in this decision-making process, this being one of the three objectives proposed in our previously published review protocol [11]. In this protocol, three objectives of interest were proposed, and due to the large amount of information gathered, it was decided to address each of the objectives separately, giving rise, so far, to two scientific articles: one focused on the participants in the decision-making process about the place of care of the elders [12] and the present article about the reasons for the place of care of the elders.

However, this study also has some limitations. A limitation of this study is that the grey literature has not been reviewed as such. However, this aspect was taken into account when planning the design of the review and the search strategy. Therefore, so it was decided to review the bibliography of the articles finally included, considering that this would provide access to the most relevant studies on the subject (whether they were part of the grey literature or not).

On the other hand, some of the studies included in this review did not report the experiences of all people directly involved in the process. Thus, sometimes we have accessed these experiences through the speech of another participant in the process in order to obtain information from all population groups of interest. This is a limitation of our results, because we have not been able to count on the direct description of all the participants, however, this decision was made with the intention of showing the various participants and the potential similarities and differences between them.

Furthermore, although some of the studies included in our review analyzed their results based on the pull and push classification, in certain cases, the results initially classified in the pull and push factors by the original authors have been reclassified by us into three factors, which implied a new interpretation. Nevertheless, this aspect, which could initially have limited our results, has finally been a strength for them, because the use of the constant comparison method has allowed to perform this process carefully; this method has required a continuous questioning of the factors and categories created and their structuring. This has generated a resulting classification and categories that have been adjusted more naturally and appropriately to the results of our review. In addition, it must be borne in mind that the literature mentions that the reasons for moving have multiple dimensions and that the well-known push and pull factors of the model of Lee are inevitably related [71].

Finally, three aspects must also be taken into account. The first is that when the review refers to the most frequently mentioned reasons, we mean that they are the ones that have appeared most often in the various articles analyzed and included in the review. That is, the frequency has been assessed by the number of studies that mention these reasons, and not by the frequency of these reasons within the studies. The second aspect to be taken into account is that, although it was chosen to analyze the interpretations provided by the authors of the studies finally included, textual quotations have also been analyzed, synthesized and extracted in certain cases due to their accuracy and importance. The third aspect we would like to point out is that the age criterion used in the inclusion criteria of this review was widely discussed among the authors, since there is currently no definition of an older person as such, and it varies according to the different societies in existence [72]. Therefore, and taking into account that our review was aimed at reviewing the international literature, with no limit per type of society or country, it was decided that the age criterion was as broad and generic as possible. In the end, the age criterion of 65 or older was chosen, since it is the criterion normally used in the literature [73,74].

## 5. Conclusions

There are many reasons surrounding the decision-making process regarding the place of care of the older people. In many cases, these reasons are consistent among the different population groups participating in the process, however, they also present interesting reasons and characteristics particular for each group. 

In addition, another important result of this review has been clustering all these reasons into a classification of three factors (retention, pull and push), which has allowed a more detailed analysis of all the present reasons reported in this decision-making process, and which, in addition, are closely related to each other. 

Thus, all this information is relevant, not only at the organizational or professional level, but also for older people themselves and for their closest environment. Fundamentally, because they will facilitate better approaches to this decision-making process, adjusting more effectively to the needs and preferences of the main parties involved in the decision. 

## Figures and Tables

**Table 1:** Characteristics of the included studies, quality assessment and relative contributions ^(a)^.

Study Country	Study Aim	Design, Methods/Techniques for Information Collection, Analysis, Sample	Assessment of Quality (AQ) Relative Contribution (RC)
Hartwigsen, 1987 [24] United States	This article discusses a relocation pattern as suggested by a sample of older widows, illuminating the identified steps taken with statements gleaned from interviews that are representative of the respondents as a whole.	Pilot study. Interviews. [Analysis process explained in detail and step by step in the article] Twenty-five older widows.	AQ: Moderate (7.5)
			RC: 25
Groger, 1994 [25] United States	This article allows the care recipients to present their own views of the decision process that resulted in their nursing home placement.	Exploratory, qualitative approach. Long interviews. [Analysis process explained in detail and step by step in the article] Ten elders.	AQ: Low (6.5)
			RC: 25
Dellasega & Mastrian, 1995 [26] [United States]	To identify and describe specific stressors experienced by family members during and after making the decision to place an elder in a skilled care facility.	Qualitative methodology. Interactive interviews. Using ethnographic summary and content analysis. Seven family members.	AQ: Moderate (8.5)
			RC: 10
Vassallo, 1995 [27] Australia	This paper shows how a therapeutic intervention was used to help an elderly couple when one of them required nursing home respite care.	[Case study] The case: a woman [older people] and her husband [family caregiver]	AQ: Low (5.5)
			RC: 23
Iwasiw et al., 1996 [28] Canada	To give voice to residents, listen to their experiences of this early phase of relocation, and thus fill a gap in the literature.	A qualitative study. Open-ended interviews. The constant comparative method of qualitative analysis. Twelve residents in long-term care facilities [older people].	AQ: Moderate (8.5)
			RC: 9
Dellasega & Nolan, 1997 [29] United Kingdom United States	To examine the positive and negative post-placement responses of family members who were involved in the placement process, and to identify possible areas for intervention.	A cross national study. Interview questions were part of a larger questionnaire [which can be consulted in other study]. Six open-ended questions were extracted from the questionnaire. Content analysis. One-hundred-and-two family members: United States: 54; United Kingdom: 48	AQ: Low (5.5)
			RC: 10
Rodgers, 1997 [30] United States	To gain information regarding specific aspects of the nursing home placement experiences of family members.	[Qualitative study]. In-depth interviews. [Analysis process explained in detail and step by step in the article] Nine family members.	AQ: High (9.5)
			RC: 37
Kao & Stuifbergen, 1999 [31] Taiwan	The research question: ‘What are the experiences of family members related to the decision of institutionalizing an elder in Taiwan?’	Exploratory qualitative study. Semi-structured interview. Content analysis. Nine family members.	AQ: Moderate (7.5)
			RC: 23
Jenkins, 2003 [32] United States	This article presents results of an exploratory study examining the care arrangement decision process from the perspective of all decision participants.	Exploratory study. In-depth, semi-structured interviews. Thematic analysis. Eleven older women; 9 family members; 5 case managers; 1 nursing home administrator.	AQ:Moderate(7.5)
			RC: 36
Park et al., 2004 [33] South Korea	To describe the experience of making the decision to place a family member with dementia in a long-term care facility among a group of 19 Korean family caregivers living in Korea.	[Qualitative study]. Semi-structured taped interviews. Thematic analysis. [Analysis process explained in detail in the article] Nineteen family caregivers.	AQ: Moderate (7.5)
			RC: 11
Caron et al., 2006 [34] Canada	To explore, from a retrospective viewpoint, the decision-making process used by family caregivers for institutionalizing a family member with dementia; and to develop a theoretical model of this decision-making process, based on the caregivers’ perspectives.	Qualitative research approach. The grounded theory method. In-depth interviews. The constant comparison of data. Fourteen family caregivers.	AQ: High (9)
			RC: 24
Groger & Kinney, 2006 [35] United States	To capture the circumstances leading up to the move, events leading to the decision to move, expectations, values guiding the decision, the actors involved in the decision, and knowledge about long-term care options.	[Mixed method study]. Qualitative face-to-face interviews. Using line-by-line text analysis. Twenty elders.	AQ: Moderate (7.5)
			RC: 34
Lynch, 2006 [36] United States	One family’s experience with elder care.	[Case report] [researcher’s mother]	AQ: Moderate (7.5)
			RC: 42
Chen et al., 2008 [37] United Stated	To develop a substantive theory of the decision-making process associated with relocation to assisted living facilities from the perceptions of elderly individuals who have moved to such setting.	Qualitative grounded theory approach. In-depth semi-structured interviews. Data were analyzed and interpreted utilizing grounded theory. Twenty-eight elders.	AQ: High (9)
			RC: 19
Kemp, 2008 [38] United States	To advance existing knowledge of later-life couples as well as life in these care settings and, in turn, inform policy and practice.	A larger, exploratory study. In-depth, semi-structured, qualitative interviews. [Analysis process explained in detail and step by step in the article] The study involved two separate, simultaneous data collection processes. Twenty married couples [older people]; 10 adult children.	AQ: Moderate (8.5)
			RC: 28
Saunders & Heliker, 2008 [39] United States	To explore the expectations and experiences of 5 newly admitted residents of an ALF [Assisted living facilities] over a 6- month period.	Qualitative exploratory design. Open-ended interview. Content analysis. [Analysis process explained in detail in the article] Five residents [older people]	AQ: High (9.5)
			RC: 7
Bekhet et al., 2009 [40] [United States]	To understand the reasons of why elders move to retirement communities and what living in retirement communities is like from the perspective of relocated elders.	Qualitative study. Interviews. Qualitative data for this analysis were collected during a quantitative study. Constant comparative method. [Analysis process explained in detail in the article] One-hundred-and-four elders.	AQ: Low (6.5)
			RC: 25
Fjelltun et al., 2009 [41] Norway	To explore carers’ and nurses’ appraisals concerning if and when NH placement for frail older people awaiting placement was needed and to illuminate ethical issues involved in deciding upon NH placement. [NH: nursing home]	Descriptive and comparative cross-sectional study using qualitative methods. Interviews. Qualitative content analysis. [Analysis process explained in detail and step by step in the article] Eleven carers with different kinship to the older people; 11 nurses	AQ: Moderate (8.5)
			RC: 35
Gottlieb et al., 2009 [20] United States	To illustrate the manner in which experiences of parents and peers are salient to older adults who may be considering health-related residential adjustments for themselves.	Exploratory study. In-person, semi-structured interviews. Case summaries were prepared from audio-recordings of the interviews. Thirty-four older adults.	AQ: Low (5.5)
			RC: 40
Jorgensen et al., 2009 [42] New Zealand	To investigate why older people entered residential care, and who was instrumental in making that decision.	A mixed method approach. Longitudinal study based on both qualitative and quantitative data. Interviews: structured and semi-structured questions. [The tools differ by group, using from interviews included with structured and/or semi-structured questions, to a standardized tool]. General Inductive approach derived from the Grounded theory. [Analysis process explained in detail in the article] One-hundred-and-forty-four older people; 47 caregivers of the older people; 12 service co-ordinators; a multidisciplinary team (four members)	AQ: Moderate (8.5)
			RC: 37
Tamiya et al., 2009 [43] Japan	To explore Japanese caregivers’ decisions to place their older family members in LTC facilities. In particular, we were interested in investigating the evidence of caregivers’ conflict during the decision-making process. [LTC: long-term care]	Case study. Content analysis was conducted on recorded family interviews. Thirty patient records: 3 cases were selected for case studies.	AQ: Low (6.5)
			RC: 23
Chang & Schneider, 2010 [44] Taiwan	To understand the Chinese family caregivers’ decision-making process of nursing home placement.	Grounded theory approach. In-depth individual interviews. [Analysis process explained in detail in the article] Thirty family caregivers.	AQ: High (10)
			RC: 12
Johnson et al., 2010 [23] United States	To identify the extent of older adults’ participation in the nursing home relocation decision and to identify the extent that SOC, functional ability, and physical functioning were related to decision-making participation. [SOC: sense of coherence]	A mixed-methods, descriptive design. Qualitative and semi-structured interviews. [Analysis process explained in detail and step by step in the article] Sixteen older adults.	AQ: Moderate (8.5)
			RC: 4
Peace et al., 2011 [45] England	To reflect the diversity of people and places in the three areas. The research sought the views of older people living in each locality.	[Qualitative study] Ethnographic methods. Nine focus groups. Fifty-four interviews. Thematic content analysis on detailed interview narratives guided by the grounded theory approach. Fifty-four respondents [older people].	AQ: Moderate (8)
			RC: 42
Tyvimaa & Kemp, 2011 [46] Finland	To identify the main attributes and features that influence the decision-making process for Finnish seniors.	Case study. In-depth interviews and open-ended survey question data. Content analysis. The three cases: Loppukiri, Kotosalla, and Keinusaari. On-hundred-and-twenty residents [older people]: 22 Keinusaari; 34 Loppukiri; 64 Kotosalla	AQ: Moderate (8)
			RC: 29
Cheng et al., 2012 [47] China	To discuss how a sample of older people and their family members made the decision to move to a specific RCF and what factors influenced the decision-making process in the socio-economic and cultural context of Beijing. [RCF: residential care facility]	[Qualitative study]. In-depth semi-structured interviews. The analysis of the data was based on the constant comparative method. [Analysis process explained in detail and step by step in the article] Twenty-seven older residents; 16 family members; 5 residential care facility managers	AQ: Moderate (8.5)
			RC: 31
Couture et al.,2012 [48] Canada	To describe the role of the health care professionals within decision-making process regarding the placement of a cognitively impaired relative.	[Qualitative study]. A grounded theory approach. Semistructured interviews. Constant comparative method. [Analysis process explained in detail and step by step in the article] Eighteen family caregivers.	AQ: High (9)
			RC: 24
Ducharme et al., 2012 [22] Canada	To build an explanatory model of the decision-making process by carrying out a prospective qualitative follow-up of family caregivers having thought about placing an elderly relative.	Qualitative grounded theory approach. Semi-structured interviews. Using ongoing comparative analysis. Eighteen family caregivers.	AQ: High (9)
			RC: 53
Söderberg et al., 2012 [49] Sweden	To reveal how family members act, react and reason when their elderly relative considers relocation to a residential home.	[Qualitative study]. Open, semi-structured interviews. A thematic analysis. [Analysis process explained in detail and step by step in the article] Seventeen family members.	AQ: Moderate (7.5)
			RC: 8
Ewen & Chahal, 2013 [50] United States	To elucidate the push-pull factors associated with moving into congregate senior housing. To investigate the perceived and experienced stresses associated with moving into a congregate living environment.	Qualitative and quantitative approaches. Phenomenological approach. Two a mixed-methods in-depth, semi-structured research interviews. [Analysis process explained in detail and step by step in the article] Twenty-six older women.	AQ: High (9)
			RC: 24
Löfqvist et al., 2013 [51] Sweden Germany	To explore how very old communityliving people reason about aging in place and relocation in very old age.	Quantitative and qualitative data. Qualitative interviews. Secondary analysis of interview data. Conventional qualitative content analyses. Eighty older people, 40 from each country.	AQ: Moderate (8.5)
			RC: 22
Söderberg et al., 2013 [52] Sweden	To reveal how the culture of independence influences the decision-making process preceding the relocation to a residential home; and since there is a predominant ideology of ageing in place, how a continued life in ordinary housing is justified versus how relocation to a residential home is excused.	[Qualitative study]. Open semi-structured interviews. Hermeneutics. [Analysis process explained in detail in the article] Twenty-one older people.	AQ: High (10)
			RC: 30
Walker & McNamara, 2013 [53] Australia	To identify key factors over different stages of relocation; to determine the range of strategies employed by older adults in relocating and maintaining a sense of home; and to explore the scope for preventative occupational therapy in promoting health and well-being throughout relocation for relatively healthy older adults.	[Qualitative study]. Semi-structured in-depth interviews. Grounded theory approach. [Analysis process explained in detail and step by step in the article] Sixteen older adults.	AQ: High (9)
			RC: 34
Wilson et al., 2013 [21] Canada	To gain an understanding of the lived experience of older patients as they wait in hospital for a nursing home bed, describe waiting placement patients and clarify their share of hospital utilization in Alberta, a western-Canadian province.	Two-part mixed method study. Interviews. Constant-comparative data analysis method. Nine older persons	AQ: High (9)
			RC: 6
Heppenstall et al., 2014 [54] [New Zealand]	To describe in-depth factors related to subsequent institutionalization and, in particular, to highlight the perceived role of social context in preventing or precipitating residential care admission.	Two qualitative methods. Telephone interviews. [Key topics from these telephone interviews were then examined in more depth with a purposively selected group of participants: the older people and the carer they chose]. Face-to-face interviews. General inductive approach. Thematic analysis. [Analysis process explained in detail in the article] One-hundred-and-forty-four older people. [Later, it was sought a purposively selected group of 15 participants. Fifteen older people and the caregiver they chose]	AQ: Moderate (7.5)
			RC: 34
Johnson & Bibbo, 2014 [55] United States	The present study used interview data immediately following the transition into a nursing home and again six to eight weeks later in order to better understand the experiences of older adults.	A larger longitudinal study. Semi-structured interviews. The analysis employed interpretive phenomenology. Thematic analysis. Eight older adults.	AQ: High (9)
			RC: 7
Koenig et al., 2014 [56] United States	To explore the older adults’ and family members’ perspectives on the decision-making process that lead up to the admission of the older adult into an AL facility. [AL: assisted living]	Qualitative study. A naturalistic paradigm. Interviews. Constant comparative method. Twenty-two older adults; 22 family members	AQ: Moderate (8)
			RC: 36
Légaré et al., 2014 [57] Canada	To determine the feasibility of implementing IP-SDM in the clinical practice of IP home care teams. To explore the perceptions of family caregivers about the decision-making process they had experienced regarding relocating their relative and about the applicability of IP-SDM in this context. [IP-SDM: interprofessional approach to shared decision making]	Exploratory case study. Individual interviews. Hybrid process of inductive and deductive thematic analysis. Six family caregivers.	AQ: High (9.5)
			RC: 7
Mamier & Winslow, 2014 [58] United States	To describe the contrasting perspectives between a family caregiver and the caregiver’s professional provider regarding the placement decision-making experience of the caregiver.	Case study. Interviews. Preliminary steps in a grounded theory. The case: One family caregiver (woman/spouse) and one professional (leader of the support group/ social worker)	AQ: High (9)
			RC: 14
Koplow et al., 2015 [59] United States	To understand the ongoing caregiving experiences of primary family caregivers during the first few months following nursing home placement of a family member.	A qualitative descriptive, two-time-point design. Interviews. [Analysis process explained in detail in the article] Ten family caregivers.	AQ: Moderate (8.5)
			RC: 11
Vasara, 2015 [60] Finland	To discuss the experiences of those who for various reasons have not chosen to or found it possible to age in place.	[Qualitative study]. Narrative interviews. [The interviews are part of a larger body of data gathered. Information explained in detail in the article] Narrative analysis. Preliminary thematic analysis. Fourteen persons [older people].	AQ: Low (6.5)
			RC: 22
Ayalon, 2016 [61] Israel	This study addresses the issue of autonomy following the transition to a CCRC, as this transition likely represents a defining point in intergenerational relations as well as in older adults’ sense of autonomy. [CCRC: continuing care retirement community]	[Qualitative study]. Interviews. [Analysis process explained in detail and step by step in the article] Thirty-six older adults; 34 family members	AQ: High (9.5)
			RC: 19
Gabrielsson-Jarhult & Nilsen, 2016 [62] Sweden	To explore older people’s concerns about their needs as expressed in discharge planning meetings at a hospital.	An explorative design based on observations was used in this qualitative study. Observations. Qualitative content analysis using both a manifest and a latent (interpretive) approach. Twenty-seven older people.	AQ: High (9.5)
			RC: 30
Nord, 2016 [63] Sweden	To explore free choice in relocation to residential care.	A qualitative study. Semi-structured, open-ended interviews. Three free will perspectives were used as the guiding theoretical framework in the analysis. Thirteen older people.	AQ: Low (6.5)
			RC: 37
Laditka, 2017 [64] United States	This study looks at the role of readiness in an older parent’s residential moves.	[Case report] [Researcher’s father]	AQ: Moderate (7.5)
			RC: 40
McKenna & Staniforth, 2017 [65] New Zealand	To gain an in depth understanding of what older people experienced during the move to residential care, the impact this had on each individual and how they coped.	Exploratory study. Semi-structured interviews. Data analyzed thematically. Nine older people	AQ: Moderate (8.5)
			RC: 9

Source: Own elaboration based on the information obtained from the 46 studies included in this systematic review. ^(a)^ The columns regarding “Author, Year of publication, Country, Study aim, Design, Methods/Techniques for information collection, Analysis, Sample” of this table have been completed respecting the information provided by the authors in the studies. In some cases, for further explanation, the authors of this review have added information that did not appear explicitly in the study, shown in the table in square brackets [].

## Supplementary Materials

The following are available online at https://www.mdpi.com/2227-9032/8/4/436/s1, Table S1: PRISMA Checklist; Table S2: Main factors: elders and family members; Text S1: Original Quotes.
