# Supplementary material for: Reasons for the Place of Care of the Elders: A Systematic Review
Source: Healthcare (Basel). 2020 Oct 27;8(4):436. doi: 10.3390/healthcare8040436 (PMC7712302; doi:10.3390/healthcare8040436)
Supplement: Supplementary file 1 [file healthcare-08-00436-s001.zip › Supplementary Material/Supplementary files. Table S2.docx]

**Supplementary material 2. Table S2: Tables of main factors: elders and family members**

**Main retention factors: elders and family members.**

| **Factor/Population** | **Categories** | **Reasons** |
| --- | --- | --- |
| Retention factors of **older people** [20,22,25,27,32,36,37,41,42,45,51,52,54,62-64] | Retention: The own house [25,27,36,41,42,45,51,52,62,64] | Wish to be at home [25,27,36,41,42,52] |
|  |  | Increase access to family members [62] |
|  |  | Postpone decision, relating it to an uncertain future and the end of life [45] |
|  |  | Maintain autonomy (freedom and privacy) [51,64] (Q1) [51] |
|  |  | Organize for independence [62] (Q2A) [62]/(Q2B) [62] |
|  |  | Maintain roles and routines [51] |
|  |  | Maintain social networks [51,64] |
|  |  | The house is a source of independence and an expression of their own identity [52] |
|  |  | Emotional bond with the home [51,64] |
|  | Rejection of other options [20,27,45,51,52,62] | Reluctance to live elsewhere [20,45,62] (Q3A) [62]/(Q3B) [20] |
|  |  | Problems arising from the move itself [51] (Q4) [51] |
|  |  | Express fear of relocation [27] |
|  |  | Negative/stereotyped opinions [45,52] |
|  |  | Reluctance to control [45] |
|  |  | Reluctance to use home help and/or technical assistance [52,62] |
|  | Facilitators [20,22,32,36,42,51,52,54,62,63] | Comfortable environment [42]; adapted house/provided equipment [20,32,42]; formal or informal help [32,36,42,54,62]; being still too healthy [63]; feeling good [22,51,52,62]; mobility of the older person [42] |
| Retention factors of **relatives**:  [22,30,32,34,36,41-43,48,49,54,59,64] | Retention: The older person's house [22,32,34,36,41-43,49,54,59] | The health status of the older person [22,41] |
|  |  | Desire to take care at home [32,36,41-43,59] Q5 [43] |
|  |  | The care was worth [54] it (Q6) [54] |
|  |  | Empathy [49] (Q7) [49] |
|  |  | Potential uprooting that could be suffered by the older person [22] (Q8) [22] |
|  |  | Loneliness they would feel if the older person was absent [22,34] (Q9) [34] |
|  |  | Difficulty in deciding for the older person [22] |
|  | Rejection of other options [22,30,32,34,41,43,48,49,64] | Feel they abandon the older person [22] |
|  |  | Unable to accept location [34,41] (Q10) [41] |
|  |  | Nursing home is the last resort [30,43] (Q11) [43] |
|  |  | Previous negative experiences regarding the quality of the place of care [41] |
|  |  | High costs of location [22] |
|  |  | Refusal of the older person [22,32,43,49,64] (Q12A) [49] |
|  |  | Refusal of professionals [48] (Q12B) [48] |
|  | Facilitators [22,34] | Positive interactions with professionals [34]; appropriate current environment [22] |

Source: Own elaboration based on the information obtained from the studies included in this systematic review.

**Main pull factors: elders and family members.**

| **Factor/Population** | **Categories** | **Reasons** |
| --- | --- | --- |
| Pull factors of **older people**:  [20,24,25,35-40,45-47,50,51,53,55,56,62,64] | Characteristics of the new place of care [20,24,25,35-38,40,45-47,50,53,56,62,64] | Comfort and location [20,24,35-37,40,45-47,50,53,56,62,64] (Q16) [35]/(Q17) [47]/(Q18) [47]: Good location [24,35,46]; services provided [20,35,46,47,50,53,56]; well communicated [20,46,47,53]; access to cultural and/or social services and/or activities [20,40,46,47,50,53,56]; proximity of family and friends [20,35-37,40,47,56,62]; staying close, familiar environment [20,35,36,40,47,62,64] |
|  |  | Social environment [20,35,45-47,50,53] (Q19A) [46]/(Q19B) [20]: Opportunities for socialization [20,35,45,46,53]; living with people of the same age [46]; associated staff [50]; characteristics of residents [47,50] |
|  |  | Familiarity and reputation [20,25,35,40,45,47,50,62] (Q20A) [40]/(Q20B) [40]: Being important, for example, having friends that are already relocated to that place [35,40,50] (Q21) [40] |
|  |  | Economy [20,37,47,50,53,56] (Q22) [47]: Financially accessible [37,47,50,53,56]; need to sell the house [56] |
|  |  | Easiness [35,36,38,56] (e.g. waiting placement, availability, being able to be in a couple, choosing a unit, taking pets, etc.) |
|  |  | Type of construction [20,35,45,46,47,50,53,56] (for example: suitability, adaptation and good aspect or appearance) |
| Pull factors of **relatives**:  [22,26,29,30-32,34,36,38,47,56,64] | Characteristics of the new place of care [22,30,31,36,38,47,56,64] | Location [30,31,36,47,56,64] (Q23) [47]: The environment [31,56]; closeness [31,36,47,56,64]: to the elder's house [36] or to the family [31,36,47,56,64] |
|  |  | The staff at these places [36,64] |
|  |  | Residents of the place of care [64] |
|  |  | Services provided [22,30,56,64] (e.g. different levels of care, specialized care, personal care, different types of therapies, etc.) |
|  |  | Appearance and/or cleaning of the new place of care [30,31,56] (Q24A) [30]/(Q24B) [30] |
|  |  | Costs/price [30,31,47,56,64] (Q25) [47]: Adjustment to the resources of the older person [47,64]; need for government aid [56]; have to sell the house of the elder [56] |
|  |  | Possibilities for testing or transferring services [36] |
|  |  | Availability of the place of care [30,31,38,56]: Besides if there is a waiting list [56]; or being able to be in couple [38] |
|  |  | Quality of the place of care [30] |
|  |  | Good connections (e.g. affiliation with a hospital) [31] |

Source: Own elaboration based on the information obtained from the studies included in this systematic review.

**Main push factors: elders and family members.**

| **Factor/Population** | **Categories** | **Reasons** |
| --- | --- | --- |
| Push factors of **older people**:  [20-25,27,28,32,35-40,42,43,45-47,50-56,60-65] | Older person's decline [20,21,23-25,27,28,32,35-40,42,45,46,50-56,60-65] | -Health deterioration [20,21,24,25,27,28,32,35,37-40,42,45,46,50,51,53-55,60,62,63] (Q27A) [40]/(Q27B) [37]  -Recognition of need for help [20,27,28,32,37,42,45,46,52,54,55,62,63,65] (Q28A) [55]/(Q28B) [37]  -Isolation/loneliness [20,24,32,40,46,53,54,61,63]  -Insecurity/unsafety [23,32,36,45,56,60,61,63,64] |
|  | The family [20,24,25,27,28,35-40,42,43,45,47,50,52,54,56,60,61,63,65] | -Feeling of burden on family and friends [20,28,35,39,42,45,47,52,54,60] (Q29A) [28]/(Q29B) [20]/(Q29C) [42]  -Death of a loved one [24,37,42,50,60,61,63] (Q30A) [42]/(Q30B) [61]  -Exhaustion of their relatives [27,36,54,56] |
|  | Formal environment [25,42,45,52,54,60,63,64] | -Professionals (due to their insistence/decision) [25,42,45,54,60,63] (Q31A) [25]/(Q31B) [42]  -The staff of the services [52,54,64]: Because of dissatisfaction with their work [52,64]; not fulfilling their needs [54]; not being able to supervise their work [52]; feeling that they cannot ask for more help [52] |
|  | Physical context [22,24,35,40,45,46,50,53,56,60,62,63,65] | -The former place of care [22,24,35,40,45,46,53,56,60,62,63,65] (Q32A) [35]/(Q32B) [60]  -The new place of care [24,40,46,50,53,60] (Q33) [40] |
|  | Economic context [24,25,32,35,50,52,53,61] | Financial concerns [24,25,32,50,52,53,61] (Q34) [53] |
|  | Inevitable, without other options [21,28,37,52-54,60,63] | -Do not have other options and do not remain any other option except to relocate [21,28,37,52,63] (Q35A) [37]/(Q35B) [21]  -Inevitable [53,54,63] |
|  | Anticipate [20,24,35,46,52,53,60,61,63] | -Prior to experienced/anticipated life changes [24,46,52,53,63] (Q36A) [24]/(Q36B) [24]/(Q36C) [53]/(Q36D) [63]  -Previous experiences of known people [20,24,35] (Q37) [24]  -Timely moment [35,53,60] |
| Push factors of **relatives**:  [22,25-27,29-34,36,38,41-45,48,54,56-59,61,63,64] | Related to the decline of the older person [22,26,27,30-34,36,38,41-45,48,54,56,58,59,61,63,64] | -Deterioration of the older person [22,26,27,30,31,33,34,36,38,41-45,48,54,58,59,61,64] (Q38A) [38]/(Q38B) [22]  -Falls [22,30,31,32,34,36,38,41,42,48,58] (Q39A) [31]/(Q39B) [22]  -Need for help of the older person [22,27,30,41,44,58,64] |
|  | Related to the caregiver [22,25,27,29,30,31,33,34,36,38,41,42,43,44,48,54,56,57,58,59,61,63] | -Unable to continue taking care [22,25,29,30,33,34,36,38,41-44,54,57,58,59,63] of : Burden of the care [22,25,29,30,33,38,41-44,54,57-59] (Q40A) [58] (Q40B) [57] (Q40C) [44]  -Health status of the caregiver [22,29,33,38,41,43,44,48] (Q41) [44]  -Absence of personal life of the caregiver [22,27,29,33,54,58,59]  -Location seen as inevitable [29,30,33,38,41,54,58] (Q42) [41] |
|  | Formal and informal environment [22,26,30,34,36,38,45,48,57,58,63] | -Opinions of the environment on that they should not continue taking care of the elder [22,26,30,38,45,58]: At the formal level [22,30] (Q43) [22]; and at the informal level [26,38,45] (Q44) [26]  -Professionals initiative [30,34,48,63]  -Pressure from professionals [22,36,48,57] |
|  | Physical context [22,34,45,56] | Physical environment is not safe for the older person [22,34] |
|  | Economic context [30,31,33,44,61] | Financial concerns [30,31,33,44,61] (Q45) [31] |

Source: Own elaboration based on the information obtained from the studies included in this systematic review.
