# Supplementary material for: Reasons for the Place of Care of the Elders: A Systematic Review
Source: Healthcare (Basel). 2020 Oct 27;8(4):436. doi: 10.3390/healthcare8040436 (PMC7712302; doi:10.3390/healthcare8040436)
Supplement: Supplementary file 1 [file healthcare-08-00436-s001.zip › Supplementary Material/Supplementary files. Text S1.docx]

**Supplementary material 3. Text S1: Original Quotes**

- Retention factors:

**ELDERS**

**Retention: The own house**

Maintain autonomy (freedom and privacy)

“Moving to a home or similar, yes? No, I don’t want that. No, No. As long as I have the strength, as long as these legs will carry me I will stay here . . . I have been down there [at the nursing home] and looked and up on north when mother staid there, and I thought uuhh, that is the final frontier . . . it is also because those moving there will not come from there alive.” [51] (p.924). Q1 [Elder]

Organize for independence

“I’m a little afraid to commit to things that are unnecessary. But we could start with a little help. I have thought about the way that I would rather work, slowly and taking it step by step. That way I can train my skills” [62] (p.139). Q2A [Elder]

“But as long as we can make it work, we think it is the best. And if we can’t manage in the house anymore. . ., we can get help with gardening and cleaning and so on. It is better than going away to a place where you might not feel comfortable” [62] (p.140). Q2B [Elder]

**Rejection of other options**

Reluctance to live elsewhere

“No, I can’t afford things. Myself and my friends who have had the home help service, they can’t afford it either and they have also declined it” [62] (p.139). Q3A [Elder]

“Because I remember my mother going to one place and saw how awful it was. I hear horror stories. I think I would rather kill myself than go into one.” [20] (p.159). Q3B [Elder]

Problems arising from the move itself

“Actually, I would [move] . . . if it was not such hard work and so expensive to move . . . I would do it. It is too big here . . . I do not need such a big flat for one person.” [51] (p.923). Q4 [Elder]

**RELATIVES**

**Retention: The older person's house**

Desire to take care at home

"No, I would like to take care of him at home as long as I can. I will try caregiving at home by using a bathing service and receiving assitance from home health aides twice a week." [43] (pp.402-403). Q5 [Relative]

The care was worth

“If she can get the help she requires and she can live the rest of her life where she wants to be and where she’s happy, that means more to me than anything else . . . her husband died in the family home and I think that’s what she wants to do . . . I think if she had a fall and it was her last fall in her own home I think she’d be the happiest woman in the world.” [54] (p.12). Q6 [Relative]

Empathy

“If it would have been me, I would not have liked to be sitting at a residential home” [49] (p.500). Q7 [Relative]

Potential uprooting that could be suffered by the older person

“We knew deep down that she wouldn’t be there long, it wasn’t the right place for her, where she’s now. But we didn’t want to yank her from her environment. . ..” [22] (pp.209-210). Q8 [Relative]

Loneliness they would feel if the older person was absent

“When you think about placing someone, there is always a fear; a fear of being alone, of finding that hard. But then, there is also the feeling that you’d be able to breathe again. I said to myself, ‘I’ll be able to sleep at night; I’ll be able to do what I want to do, not just what he wants to do’. But I kept it up because I just couldn’t imagine myself being all alone, even though it was really difficult with him at home. I just couldn’t imagine living with the fact that he was in a nursing home. I even refused the first time they called me to say that a room was available.” [34] (p.201). Q9 [Relative]

**Rejection of other options**

Unable to accept location

“As long as I can manage, I want him to be home…. We have been married for almost 50 years and we have had a very good life” [41] (p.3083). Q10 [Relative]

Nursing home is the last resort

"...special nursing homes, and long-term care hospital for older adults are the last resort." [43] (p.403). Q11 [Relative]

Refusal of the older person

“That I felt you know, when she had said no, that there went that freedom away [laughs]. Yes, that I got to say, that I would have been relieved if she had moved there, so that everything would calm down for me (‐‐‐). I have to accept her choice, you know.” [49] (p.499). Q12A [Relative]

Refusal of professionals

“The doctors administered many tests. They concluded that my mother was able to go back home. I didn’t think it was appropriate. She passed the tests, but I knew she wasn’t able to go back home” [48] (p.287). Q12B [Relative]

**PROFESSIONALS**

**Family support**

“could never have agreed to this arrangement if it weren’t for her daughters living so close.” [32] (p.137). Q13 [Professional]

**Satisfy the family**

“I never thought when I first saw Mrs. H. that she would go home . . . [she was] so sick. She could barely get in and out of bed by herself . . . couldn’t walk more than a few steps even with a walker. Her daughters were just insisting that she had to go home . . . a nursing home would ‘kill her.’ But they couldn’t give her enough help. We finally put together enough services so that we agreed to try letting her go home . . . So far it’s working okay, but if she falls again when she’s alone I don’t know . . . it’s only because of her daughters she can manage now.” [32] (p.138). Q14 [Professional]

**Other relevant people**

**Older people were too young to move**

“the timing’s not right, the timing’s not right, it’s too soon.” [35] (p.84). Q15 [Other relevant people (Friend of the older person)]

- Pull factors:

**ELDERS**

**Characteristics of the new place of care**

Comfort and location

“If you want to go someplace you can lock up and go. You don’t have to worry about how somebody’s got to cut the grass, somebody’s got to watch the place, you know, it’ll be taken care of . . . I got to the point I thought I don’t want to climb to put light bulbs in. I had to put a light bulb in upstairs and I had to get a chair. It was in the middle of the room and the fixture is a heavy one and I called my brother I said I’m going to put a light bulb in. I’m climbing up on a chair. I said if I don’t call you back in 20 minutes, you call me or else come.” [35] (p.95). Q16 [Elder]

“(…) I can get to my appointed hospitals easily." [47] (p.1284). Q17 [Elder]

"I chose this RCF because it locates close to where my children live. That is easier for them to come to visit.(…)" [47] (p.1284). Q18 [Elder] [residential care facility]

Social environment

“One of the most important things that attracted me was the idea of a house where everyone knows each other—that it’s like one big family. And that expectation has been met.” [46] (p.64). Q19A [Elder]

“you are socially connected” [20] (p.160). Q19B [Elder]

Familiarity and reputation

“I was familiar with [name of the facility]. . . . My aunt was a former resident, you know, the facility has a good reputation.” [40] (p.468). Q20A [Elder]

“[I was] so familiar with [name of the facility] . . . it was not a problem at all to move here, I mean, I feel like I’m at home. . . . [It’s a] nice feeling to have . . . you know . . . and to live with. . . .” [40] (p.471). Q20B [Elder]

Familiarity and reputation: Having friends that are already relocated to that place

“I have friends here. . . . We all went to school together . . . graduated at the same time . . . and shared every single moment together. . . . It was nice and fun joining them here. . . . I mean, you feel that they are part of your past and . . . your future as well.” [40] (p.471). Q21 [Elder]

Economy

“My pension is a little more than 1,000 Yuan per month . . . I moved to a single roomat the beginning, but my son persuaded me to switch to a three-bed room a month later, because the single room is twice as expensive as the shared three-bed room.” [47] (p.1287). Q22 [Elder]

**RELATIVES**

**Characteristics of the new place of care**

Location

"I live very close to here. I can stop by several times a day, on my way to work and way back home. I always drop by to talk to him [the interviewee’s father] for a while, check if everything is alright, and bring him some of his favourite dishes . . . I won’t be able to do so if he lives in a RCF far from me, (in that case) maybe I can only visit him twice a month.” [47] (pp.1284-1285). Q23 [Relative] [RCF: residential care facility]

Appearance and/or cleaning of the new place of care

"I would always ask to see the kitchen. I always think the kitchen tells the story. If it's clean in the kitchen, it's clean." [30] (p.60). Q24A [Relative]

“A lot of [the residents] have bedsores, because they'd be sitting there all day wet. You can come in and smell it. Usually, I go by what I smell in a nursing home. When I first walked into [the nursing home chosen] I was pleased. It didn't have that smell.” [30] (p.60). Q24B [Relative]

Costs/price

“My mother-in-law does not have pension. Her four children split all the expenses, including her living expenses and health-care expenses . . . For some upscale RCFs, we cannot even think about it because of the high expenses.” [47] (p.1287). Q25 [Elder] [RCFs: residential care facility]

**Information on places of care**

“I had no qualm about putting her here because everything I had heard, and people I had talked to. I have friends that have family members here, a girl that works for me, her father is here. Everyone told me that they get good care.” [26] (p.132). Q26 [Relative]

- Push factors:

**ELDERS**

**Older person's decline**

Health deterioration

“My physical disability is the reason. . . . [I was] unable to live alone because of the stroke, I needed assistance.” [40] (p.467). Q27A [Elder]

“I was not able to, at my age, do all the yard work, repairs and things like that. It became one of the problems.” [37] (p.93). Q27B [Elder]

Recognition of need for help

“I knew I needed help,” [55] (p.60). Q28A [Elder]

“I moved here to have somebody close by in case I needed assistance. I don’t need assistance now but I might someday.” [37] (p.98). Q28B [Elder]

**The family**

Feeling of burden on family and friends

"This place was my first choice right from the heart...this is what I wanted. I wanted to come here because I didn´t want my children to be burdened with my problems." [28] (p.384). Q29A [Elder]

“But it changes your life, and I don’t want to be the catalyst for any changes in my kids’ lives.” [20] (p.157). Q29B [Elder]

“not wanting to be a burden” [42] (p.25). Q29C [Elder]

Death of a loved one

“I felt that my health was getting worse and I couldn’t manage to do the things after my husband died, so I thought that this [residential care] was the best place for me” [42] (p.24) Q30A [Elder]

“he (husband), died. I stayed all alone. I told myself, ‘how long can I stay like this, All alone.’ I was really afraid to stay home alone. At 6 p.m., I already shut everything off.’’ [61] (p.138). Q30B [Elder]

**Formal environment**

Professionals (due to their insistence/decision)

“The doctor didn’t want me to live alone, and it was his suggestion that I come into a nursing home. Really, it is his decision. One of the grandchildren consented to stay with me, but then there were too many hours in between their working hours and the doctor felt I shouldn’t be at home alone that long. So he said, ‘Well, I think it would be better for you to go into a nursing home.’ So I said, ‘Well, okay, if it can’t be helped.’(…)” [25] (p.88). Q31A [Elder]

“The nurse from the rest home [residential care]came over to my unit and said that since I was incontinent that I should not be here; I should be in [residential care] [and that] I was not able to look after myself when I was incontinent” [42] (p.24). Q31B [Elder]

**Physical context**

The former place of care

“I have two lots here and that’s almost an acre and cutting the grass and then my knee was getting worse, you know, the osteoarthritis and that. I was in pain all the time and limping, you know, so I thought, gee I just can’t handle all this and we had hedges on each end and I had to cut those. My nephew would help me and my brother would help me, but you know, they have their places to take care of and you just hate to constantly ask them.” [35] (p.88). Q32A [Elder]

“(…) Sure, I would've liked to live there, but it, it was on the outskirts, and there was no…. I couldn't drive a tractor and a tractor would've been necessary to plough the roads, so that you could get out and about. I had never learned how to do it, although I was a farmer. Like that, that's the way it was. It really got to me, but when you can't, you can't.” [60] (p.60). Q32B [Elder]

The new place of care

“[It] seems like a sensible thing to do . . . a place where I can live with less responsibility, others can do stuff for me.” [40] (p.469). Q33 [Elder]

**Economic context**

Financial concerns

“And so then I thought ‘here am I in this big house’ and it’s all getting expensive because you’ve got to pay for a lot of help. It was beginning to need things, like [fixing] rusty gutters. So I looked around, and I found this place advertised…I made enquiries here and they had quite a lot available then. So I had a big choice.” [53] (p.448). Q34 [Elder]

**Inevitable, without other options**

Do not have other options and do not remain any other option except to relocate

“My son helped me sell my home when I was in the nursing home for four months after my hip replacement. after my hip replacement. He said I could not stay at home alone and nobody could be home with me. I knew I could not stay at my daughters’ places. They have their own things you know. I was not able to live at home. There was no place back home that I could stay. So I could not stay there, so I moved here.” [37] (p.98). Q35A [Elder]

“what can I do?" [21] (p.17). Q35B [Elder]

**Anticipate**

Prior to experienced/anticipated life changes

“Knowing that I wasn’t going to be able to drive I had to choose a place where transportation would be available, where services . . . food . . . and where friends would be available. And a place where I could learn to be comfortable in before I lost as much sight as I would be losing (spoken by a respondent affected by macular degeneration).” [24] (p.201). Q36A [Elder]

“. . . I was going (to the life care facility) where I would be planning my future. I knew that out here if I got sick, they had the health center to take care of me.” [24] (p.201). Q36B [Elder]

“And so then I thought ‘here am I in this big house’ and it’s all getting expensive because you’ve got to pay for a lot of help. It was beginning to need things, like [fixing] rusty gutters. So I looked around, and I found this place advertised…I made enquiries here and they had quite a lot available then. So I had a big choice.” [53] (p.448). Q36C [Elder]

“It wasn't particularly difficult to choose what to bring [here]. It [the move] wasn't sudden, you know. I could drag it out because we had agreed that I should move.” [63] (p.62). Q36D [Elder]

Previous experiences of known people

“My husband and I on our trip to Memphis . . . had started discussing this type of living (life care). We had seen friends that were living and needed help and weren’t getting it. They were no longer able to take care of themselves and were still living in a single home and were dependent on neighbors and friends and we didn’t want that to happen to us.” [24] (p.202). Q37 [Elder]

**RELATIVES**

**Related to the decline of the older person**

Deterioration of the older person

"It was getting costly to have these caregivers coming into the house. And you know, you have to fire them and hire new ones and when they’re in their own house, you have to watch what’s going on. . . . We all had this conversation off and on really for about a year before I made a decision that this was it. I said, ‘Y’all, this is it. We have to go. I’m the one taking care of things.’ . . . They had a beautiful home and they both wanted to stay there, but they knew it was time. They were both accepting of it.” [38] (p.239). Q38A [Relative]

“We could tell that she was more confused. Often, she had burn marks on her arms. She had these marks and she didn’t know how she got them” [22] (p.205). Q38B [Relative]

Falls

“She fell a couple of times at home. Her head was injured and her right arm was broken. We worried that she might be in danger if she kept falling like this. She needed somebody to keep an eye on her every minute. We couldn't do that for 24 h a day so we sent her here.” [31] (p.1121). Q39A [Relative]

“Of course, after the fall, there and then, it was clear that she would not be going back to where she had been living” [22] (p.205). Q39B [Relative]

**Related to the caregiver**

Unable to continue taking care: Burden of the care

“but I’ve reached the point that I don’t think I am physically able. . .” [58] (p.15) Q40A [Relative]

“On the week-end it was like I was in jail… without bars but I was in jail” [57] (p.7). Q40B [Relative]

"I don’t know how to take care of my wife because she always tosses and turns from side to side. She can’t control urination and bowel movement. Last time, she got a big bedsore on the buttock. So, I need their [professional] help to take care of her." [44] (p.112). Q40C [Relative]

Health status of the caregiver

"I have major depression. . . . I do not want to give him the shots [for diabetes] and I do not want to test blood sugar for him, either. . . . He asks me many questions that make me feel frazzled. So, I just do my duty- giving him the medication and cooking. After that, I just hide in my room . . . My current situation may be harmful to him." [44] (p.112). Q41 [Relative]

Location seen as inevitable

“When we get an offer of NH placement, we cannot refuse it. Although she has always told us she will die before moving to a NH, she will just have to accept it” [41] (p.3084). Q42 [Relative] [NH: nursing home]

**Formal and informal environment**

Opinions of the environment on that they should not continue taking care of the elder: At the formal level

“I’d prefer to keep her a little while longer in order to get her into a place like that. But the doctor would like me to place her because he says: ‘A lot of your energy is being spent taking care of her. It’s taking a toll on you. Sooner or later, you’re the one who’s going to need help getting up. You can’t afford to ruin your own health.’” [22] (p.205). Q43 [Relative]

Opinions of the environment on that they should not continue taking care of the elder: At the informal level

"I thought about bringing him to the house but everybody told me that was wrong. Because they said that you don't realize what you're in for. They said that you can't handle it." [26] (p.131). Q44 [Relative]

**Economic context**

Financial concerns

“Hiring an aide at home cost us a lot of money every month. Almost half of our salary was gone. Compared to hiring an aide, a nursing home is cheaper.” [31] (p.1121). Q45 [Relative]
